# Immunomodulatory plasticity of mesenchymal stem cells: a potential key to successful solid organ transplantation

**DOI:** 10.1186/s12967-018-1403-0

**Published:** 2018-02-15

**Authors:** Urvashi Kaundal, Upma Bagai, Aruna Rakha

**Affiliations:** 1 Department of Translational and Regenerative Medicine, Postgraduate Institute of Medical Education and Research, Sector 12, Chandigarh, India; 2 Department of Zoology, Panjab University, Sector 14, Chandigarh, India

**Keywords:** Organ transplantation, Graft survival, Mesenchymal stem cells, Microenvironment

## Abstract

Organ transplantation remains to be a treatment of choice for patients suffering from irreversible organ failure. Immunosuppressive (IS) drugs employed to maintain the allograft have shown excellent short-term graft survival, but, their long-term use could contribute to immunological and non-immunological risk factors, resulting in graft dysfunctionalities. Upcoming IS regimes have highlighted the use of cell-based therapies, which can eliminate the risk of drug-borne toxicities while maintaining efficacy of the treatment. Mesenchymal stem cells (MSCs) have been considered as an invaluable cell type, owing to their unique immunomodulatory properties, which makes them desirable for application in transplant settings, where hyper-activation of the immune system is evident. The immunoregulatory potential of MSCs holds true for preclinical studies while achieving it in clinical studies continues to be a challenge. Understanding the biological factors responsible for subdued responses of MSCs in vivo would allow uninhibited use of this therapy for countless conditions. In this review, we summarize the variations in the preclinical and clinical studies utilizing MSCs, discuss the factors which might be responsible for variability in outcome and propose the advancements likely to occur in future for using this as a “boutique/personalised therapy” for patient care.

## Background

Over recent years tremendous progress has been made to understand the basic mechanisms underlying the state of allograft rejection. Regardless of substantial improvements in short-term allograft survival, long-term outcome remains subpar [1–4]. The current maintenance regimen to support organ transplantation and to reduce transplant-related morbidity includes a combination of immunosuppressive (IS) drugs including calcineurin inhibitors, mTOR inhibitors and anti-proliferative agents [5]. Application of IS drugs has a therapeutic and suppressive effect on host’s immune system. Nevertheless, non-specific immunosuppression produced by IS drugs, also result in instances of undesired immunodeficiency, toxicity to other non-immune cells, cardiovascular disorders and malignancies [6–11]. In the last decade, extensive research in the field of translational medicine has indicated the use of cell-based therapies complementary to IS drugs for achieving the goal of ultimate IS therapy i.e. a therapy that can induce a balance between maximum efficacy and minimal adverse effects.

Mesenchymal stem cells (MSCs), have recently gained the interest of clinicians and researchers. The likelihood of these MSC based therapies depends upon, their regenerative facets and modulation of the immunological responses engendered through their secreted paracrine mediators [12]. MSCs are recognized for the activation of regulatory immune cells in conjunction with interference in maturation and activation of antigen presenting cells (APCs). As already known, exogenously cultured MSCs upon administration into the patient’s body, interact with the microenvironment in vivo which leads to their activation or licensing. Clinical studies have suggested that this licensing process in vivo is mediated by the presence of soluble factors and cytokines in the circulation. MSCs upon exposure to different concentrations of inflammatory mediators either produce Th1 or Th2 cytokines, growth factors, cell migration factors which assist in tissue maintenance and repair. Along with the inflammatory cytokines, other factors like in vitro culture conditions, Toll-like receptor (TLR) signalling and drug interactions in vivo, may also determine the clinical efficacy of MSCs.

This review aims to describe the influence of microenvironment both in vitro and in vivo on MSC and their implications on various preclinical and clinical studies.

## Mesenchymal stem cells—physical and functional profile

Mesenchymal stem cells originally reported by Friedenstein et al. [13, 14], are multipotent progenitor cells accomplished to differentiate into several specialized cell types. At high density, MSCs, align with each other in a typical spatial pattern and have spindle-shaped fibroblastoid morphology [15]. MSCs righteously referred to as mesenchymal stromal cells, possess trans-differential potential, triggered by, placing MSCs under specific stimuli which advance their development into various lineages namely mesodermal i.e. myocyte, adipocytes, osteocytes, cardiomyocytes, endothelium; ectodermal i.e. neuronal; and endodermal i.e. hepatic, respiratory, pancreatic epithelium [16–18]. Bone marrow (BM) is considered as a primary source of MSCs while other sources include adult connective tissues such as dental pulp, peripheral blood, adipose tissue and foetal tissues such as Wharton’s jelly, placenta, amniotic fluid, umbilical cord (UC) and umbilical cord blood [19].

Phenotypically, MSCs are recognized by expression of surface markers CD105, CD73, CD90 (mesenchymal lineage markers) and lack of expression of CD34, CD19, CD45, CD11a (hematopoietic lineage markers), CD31 (endothelial lineage marker), HLA-DR (human leukocyte antigen) [18].

Mesenchymal stem cells express intermediate levels of class I major histocompatibility complex (MHC) and do not express class II MHC [18, 20] or other co-stimulatory molecules like B7-1, B7-2, CD80, CD40, CD40L or Fas ligand on their surface [21], which play a crucial role in immune activation. Even though the expression of MHC-II molecules on MSCs is upregulated when stimulated with a low-dose of pro-inflammatory cytokine—interferon (IFN)-γ, no modification in the expression of co-stimulatory molecules is observed [21, 22]. This peculiar profile of MSCs, makes them immune evasive and thus an attractive candidate for cell-based therapies for various clinical conditions.

## Immunological tolerance and mesenchymal stem cells

During transplant rejection, graft from a genetically different donor elicits an allogeneic immune response inside recipient’s body, generated against antigens present on donor graft. The allogeneic immune response is a consequence of an intricate sequence of interactions involving both innate (dendritic cells, macrophages, neutrophils, mast cells and natural killer cells) and adaptive (T and B cells) immune system, ultimately leading to rejection and transplant failure [23–25]. In order to manage early graft rejections, IS drugs are administered but their effect on controlling long-term graft rejections is questionable [26]. Keeping this in mind, the clinical emphasis recently has shifted towards induction of a state of tolerance towards an organ allograft [27, 28]. Transplantation tolerance is a state marked by the absence of donor-specific inimical immune responsiveness which can be maintained devoid of chronic immune suppression [29, 30]. Although transplant tolerance has been successfully achieved in various animal models, its accomplishment in humans remains incomprehensible [31–33]. Insights into mechanisms involved in immune activation have led scientists to evaluate cell-based therapies specifically using MSCs for various disease conditions owing to their powerful immunomodulatory potential, without any long-term deleterious effects [34, 35].

Existing data substantiates that MSCs help in instigating a state of tolerance by suppressing the effector cell responses that pose a major threat to the transplanted organ [36]. MSCs possess broad immunoregulatory properties which can modulate the immune responses by strongly inhibiting the differentiation, maturation, function and proliferative responses of immune cells both in vitro and in vivo [37–39]. Studies have demonstrated the potency of MSCs to induce regulatory T (Tregs) and regulatory B (Bregs) cell activity which further directs suppression of effector and memory immune cell responses [40]. Moreover, MSCs are capable of inhibiting the generation and maturation of dendritic cells (DCs), which impairs their capacity to activate T cells [41]. MSCs can do so by inducing tolerogenic DCs, which produce interleukin-10 (IL-10) and result in an expansion of Treg cells through an indirect mechanism [42]. Similarly, MSCs can also alter the macrophage phenotype from a pro-inflammatory phenotype M1 to an anti-inflammatory phenotype M2 and lead to the generation of MSC-educated macrophages (MEM) possibly using an IL-10 mediated switch [43]. Interestingly, in response to a pathogenic insult, MSCs enhance the microbicidal activity of macrophages by altering the naive macrophages into the inflammatory M1 macrophages, without enhancing their APC function. However, MSCs induce the conversion of already active M1 macrophages into anti-inflammatory M2 macrophages to resolve the hyper-inflammatory state [44]. MSCs have also been shown to suppress the IL-2 mediated proliferation and cytotoxic activity of Natural Killer (NK) cells [45]. These aforementioned immunosuppressive properties inflicted by MSCs possibly lead to induction of a state of peripheral tolerance [46, 47].

For contact-dependent mechanisms, MSCs express a large number of chemokines which lead to chemotaxis of immune cells in the close vicinity of MSCs. As soon as immune cells begin to migrate, MSCs secrete locally active immunosuppressive factors which act on these migrating immune cells in a reciprocal fashion [48]. While paracrine mechanisms are conceded by MSCs through direct secretion of anti-inflammatory factors such as transforming growth factor (TGF-β), hepatocyte growth factor (HGF), nitric oxide (NO), heme oxygenase (HO)-1, indoleamine 2,3-dioxygenase (IDO) and expression of inhibitory co-stimulatory molecules such as TNF-related apoptosis-inducing ligand (TRAIL), programmed death ligand (PD-L1) that work together and influence the effector populations [49]. Through indirect mechanisms, MSCs can either inhibit the maturation of the antigen presenting cells (APCs) or generate regulatory or suppressor cell populations through paracrine signalling [50]. Paracrine signalling pathways are thought to be crucial as MSCs themselves are short-lived due to their susceptibility to lysis by CD8^+^ T cells and NK cells [51, 52]. Interestingly, a recent study has indicated the “apoptotic demise” of MSCs as a key step for activation of effector mechanisms of immunosuppression lead by MSCs [53].

Although a number of factors are known that help in MSC-mediated immunomodulation but every factor serves a different function depending upon the MSC source and its microenvironment. Therefore, further understanding of how MSCs can be directed to produce the “beneficial factors” and how these factors can regulate immune cells, might lead to the achievement of a state of tolerance/partial tolerance through immunomodulation.

## Inconsistency amongst the preclinical and clinical findings using MSCs

Theoretically, the idea of utilizing MSCs as an adjunct immunosuppressive therapy for solid organ transplantation is very alluring as MSCs aid in stimulating tolerogenic immune responses. To test this hypothesis, plenty of preclinical studies in experimental models of organ transplantation have been conducted. The first preclinical study was performed by Bartholomew et al. [54] in a baboon model of skin transplant which illustrated a diminished lymphocyte proliferation with an enhanced graft survival as a result of intravenously infused allogeneic MSCs. Further, studies performed in different experimental transplant models demonstrated that MSC infusion holds the potential to prolong graft survival with minimal rejection rate and this occurred either through suppression of effector T cells or amplification of regulatory cell subsets or both [55–60]. The data from these preclinical studies have established safety and efficacy of MSCs as a complement to IS therapy which has encouraged their clinical application in patients undergoing transplantation. Till date, over 734 clinical trials investigating the effectiveness of MSC therapy in different immune-mediated or related conditions have been registered on the clinical trial database (clinicaltrials.gov). Using keywords “mesenchymal stem cells” and “transplant”, we were able to find 253 registered trials and out of these only 12 clinical trials are being conducted in solid organ transplant patients. All of these studies are still in Phase 1 or combined Phase1/2 and are dedicated to evaluate safety and efficacy of MSC therapy (Table 1). The first pilot study reporting safety and feasibility of autologous MSC therapy for kidney transplant patients with living related donors surfaced in 2011 [61]. In the following year, Tan et al. [62] demonstrated that autologous MSC infusion in transplant recipients lead to decreased frequency of allograft rejection and opportunistic infections as compared to the control group given anti-IL-2 receptor induction therapy. However, the rejection rates and renal function outcome in MSC treated patients and control group patients were similar at 6 months. Further, a study conducted by Reindeers et al. [63] revealed that a decrease in donor-specific proliferation of peripheral blood mononuclear cells (PBMCs) of MSC-treated patients, 12 weeks post-infusion, but the incidence of viral infections was higher in these patients. Regardless of substantial clinical data, which proved the safety of MSC therapy, there were no considerable supporting reports to confirm the immunological status of treated patients. As the immune profile of patients is the key to foresee the short and long-term graft outcome, subsequent studies focussed on monitoring of both clinical as well as immunological parameters. A pilot study by Peng et al. used a combination of allogeneic MSCs and a low dose of tacrolimus (TAC) to prevent renal allograft rejection [64] and immune profile of these patients was regularly monitored till 1-year post-transplant. The authors remarked the use of allogeneic MSCs as safe and feasible as no incidence of acute rejection was evident in the experimental group. While comparing immune profiles, only a slight change in the percentage of B cells at 3 months post-transplant was observed while there was no change in CD4/CD8 T cells, NK cells or intracellular cytokine expression. Other studies have reported that MSC infusion in a transplantation setting has a Treg cell promoting effect which in return weakens memory and effector T cell responses [61, 65]. A majority of documented studies have explored the use of MSCs in kidney transplant patients and autologous BM aspirates have been used as a preferable MSC source. Although studies in experimental models have indicated that MSCs of both autologous and allogeneic origin display the same immunosuppressive potential still the decision of whether to use donor-derived MSCs or not needs powerful consideration. Allogeneic MSCs have been shown to have enhanced immunogenicity in vivo which might lead to an anti-donor immune response [66, 67]. For the same reason, very few studies (n = 2) have employed allogeneic MSC infusions for patient trials [64, 68].

**Table 1:** Clinical trials related to use of MSCs for organ transplantation

Organ transplanted	MSC source	MSC dosage	IS regime	Route of administration	Patients enrolled/ estimated enrolment	Trial phase	Status
Kidney	Autologous Bone marrow-derived MSCs (BM-MSCs)	Doses for different groups 2 × 10^6^ MSCs/kg, 48 h before Tx 2 × 10^6^ MSCs/kg + 5 × 10^6^ cell, 48 h before Tx 2 × 10^6^ MSCs/kg at day 1, day 7	Anti-thymocyte globulin (ATG), Prednisolone, Mycophenolate mofetil (MMF), Calcineurin inhibitor (CNI), IVIG ATG Plasma exchange, IVIG, anti-CD20 monoclonal antibody	Intravenous (I.V.) or intra-arterial (I.A.) I.V.+ I.A. I.V.	260	1	Enrolling NCT number-NCT02490020
Liver	Umbilical cord Derived MSCs (UC-MSCs)	Multiple doses: 1 × 10^6^ MSCs/kg body weight for 12 weeks (once per 4 weeks)	Not mentioned	I.V.	50	1	Unknown NCT number-NCT01690247
Kidney	Third party BM-MSCs	Four doses: 1 * 10^6^/kg every week	Not mentioned	I.V.	120	1/2	Ongoing NCT number-NCT02563366
Kidney	Autologous, BM-MSCs	Single dose 2 × 10^6^ MSCs/kg body wt given 1 day before Tx	MMF, Tacrolimus (TAC) CsA, Steroids	I.V.	159	2	Completed NCT number-NCT00658073 [62]
Kidney	Autologous, BM-MSCs	Two doses: 1–2 × 10^6^ MSCs/kg during Tx and day 7 post Tx	MMF, TAC or CsA, Prednisolone	I.V.	15	1	Completed NCT number-NCT00734396 [63]
Kidney	Autologous, BM-MSCs	Single dose 2 × 10^6^ MSCs/kg body wt given 1 day before transplantation (Tx)	ATG, MMF, Cyclosporine A (CsA), Steroids	I.V.	4	1/2	Terminated NCT number-NCT00752479 [69]
Kidney	Allogeneic, BM-MSCs	Two doses: 1st dose of 1–2 × 10^6^ cells/kg 1 day before Tx, 2nd dose- 1–2 × 10^6^ cells 30 days after Tx	TAC, MMF, Prednisolone	I.V.	15	1	Active, not recruiting NCT number-NCT02409940
Kidney/liver	Allogeneic, BM-MSCs (Third party)	Single dose of 1.5–3 × 10^6^ MSCs/kg at 3 (± 2) days after Tx	TAC, MMF, Steroids	Not mentioned	40	1/2	Unknown NCT number-NCT01429038
Kidney	Allogeneic, BM-MSCs (Third party)	4 doses: 1 × 10^6^ cells/kg during Tx and at day 7, 14, 21 post-Tx	Induction with basiliximab, Low dose of TAC, MMF, Steroids	I.V.	120	1/2	Not yet recruiting, NCT number-NCT02561767
Kidney	Autologous, stromal vascular fraction derived MSCs	4 doses: 1 × 10^6^ cells/kg during Tx and at day 7, 14, 21 post-Tx	Basiliximab induction	Not mentioned	120	1/2	Not yet recruiting, NCT number-NCT02492308
Liver	Allogeneic, BM-MSCs	Two doses: 1 × 10^6^ MSCs/kg during Tx and day 2 (± 1) post-Tx	TAC, Basiliximab, Steroids	1st dose: intraportal infusion, 2nd dose: I.V.	7	1 (Pilot study)	Recruiting NCT number-NCT02957552
Kidney	Allogeneic, BM-MSCs	Two doses: First dose of 5 × 10^6^ cells/kg during Tx, Second dose of 2 × 10^6^ cells/kg 30 days after Tx	Cytoxan, Methylprednisolone (MEP), Low dose of TAC, MMF	1st dose: renal allograft artery, 2nd dose: I.V.	12	Pilot study	Completed [64]
Kidney	Allogeneic, BM-MSCs	Two doses: First dose of 5 × 10^6^ cells/kg during Tx, second dose of 2 × 10^6^ cells/kg 30 days after Tx	Cytoxan, MEP, Low dose of TAC, MMF	1st dose: renal allograft artery, 2nd dose: I.V.	32	Pilot study	Completed [68]
Kidney	Adipose-MSCs and BM-HSCs	0.03 × 10^6^ MSCs/kg + 8–10 × 10^8^ HSCs per kg 5 days before Tx	ATG, Total lymphocyte irradiation, TAC, MEP	Portal infusion	285	2	[70]

Data from clinical trials have repeatedly highlighted safety and tolerance of MSCs in humans but given the current scenario, it is difficult to state the long-term therapeutic efficacy of MSCs. Small sample size, the primary cause of the disease in a patient, treatment regime, difference in the immune cell and cytokine profile which decide the effectiveness of a treatment course are some of the factors which make the translation of preclinical findings challenging in human subjects. Moreover, follow-up for a limited time period, different efficacy endpoints and insufficient cellular and molecular findings are some factors which make it difficult to infer anything concrete from these studies.

## Microenvironmental cues influencing therapeutic efficacy of MSCs

Although immunosuppression by MSCs is a well-documented notion, its mere understanding does not guarantee a successful outcome in vivo. In vitro studies provide a better control as they allow close monitoring of MSC fate. While after in vivo administration, MSCs become exposed to host immune cells and soluble cytokine/chemokine mediators which modulate their phenotype, thus indirectly controlling their fate, which can either impact the outcome positively or negatively. During disease progression, the role of cytokines is domineering in the acute phase of inflammation. But the inflammatory profile of patients is not stable and it varies from time to time at different stages of disease pathogenesis. These fluctuating cytokine profiles are responsible for the incompetence of MSC therapy in preclinical and clinical studies. Moreover, the drugs used for managing organ transplants and immune-mediated disorders are mostly immunosuppressive in nature which further add to the complexity. The concept of MSC microenvironment has gained the significant attention of the researchers worldwide and may serve as the key element for deciding the success of stem cell therapy. To adapt these cells effectively for clinical applications, in this review we have enlisted relevant factors/conditions which have already been indicated to affect the potency of MSCs.

### Oxygen conditions

Maintenance of appropriate oxygen concentration in vitro is important for anticipating therapeutic efficacy of MSCs.

Mesenchymal stem cells in vivo reside in perivascular niches in close association with blood vessels in nearly all the tissues. Although MSCs reside near microvasculature, yet, the various tissues where they are located exhibit depleted levels of oxygen. The oxygen concentration in MSC niche is about 2–8% that is almost half of the oxygen tension in arterial blood [71, 72].

Along with this, oxygen pressures experienced by different tissues from where MSCs can be isolated are variable, i.e. 1–7% for bone marrow, 15% for adipose tissue and 1.5–5% for reproductive tract and birth-associated tissues [73].

Mammalian cells are cultured in vitro at 21% O_2_, which is considered normoxic according to conventional standards set in cell culture practice. These non-physiological culture conditions expose these cells to approximately 10 times the concentration of O_2_ which they normally encounter in vivo. However, recently it has been established that lower oxygen concentration is crucial for maintaining the undifferentiated state of MSCs and can also influence their proliferation rate and cell-fate commitment [74, 75]. Studies have revealed Hypoxia-inducible factor (HIF) pathway as the crucial signalling pathway which gets activated in MSCs when cultured in low oxygen conditions [76]. HIF-1 alpha (HIF-1α) and HIF-2 alpha (HIF-2α) are the key molecules which have protective effects on MSCs and help them in promoting cellular adaptation in response to hypoxic condition [77–79].

Hypoxia or physiological normoxia leads to an enhanced immunomodulatory potential of MSCs. Researchers have reported an increase in anti-inflammatory cytokine production of MSCs culture in response to hypoxia [80, 81]. Human MSCs cultured in hypoxic conditions demonstrate a decrease in differentiation capacity and high expansion rate when compared to MSCs cultured in normoxia which is indicative of maintenance of the multilineage potential of these cells [82, 83]. Moreover, MSCs cultivated under hypoxic conditions exhibit superior genetic stability [84] and decreased apoptosis [85] which under normal oxygen concentration can induce oxidative stress leading to the production of reactive oxygen species (ROS) that damages the cellular DNA and proteins [86]. Collectively, hypoxia can conserve the primitive properties and enhance the immunoregulatory functions of MSCs [87–89] which can be beneficial for their clinical applications Table 2.

**Table 2:** Preclinical studies related to preconditioning of MSCs

Cell source	Preconditioning	Experimental model	Factors affected	Implication	References
Human BM-MSCs	Hypoxia	GvHD (mice)	Increase in stemness factors: Kruppel like factor 4 (KLF4), Octamer-binding transcription factor 4 (OCT4), v-myc avian myelocytomatosis viral oncogene Homolog (C-MYC), Increase in chemokine genes- CCL2, and CXCL10	Enhanced chemotaxis, viability and homing	[90]
Murine BM-MSCs	Hypoxia	Cellular cardiomyoplasty	Increase in anti-inflammatory cytokine expression as compared to pro-inflammatory cytokines	Improved cardiac function	[91]
Human BM-MSCs	Hypoxia	Limb ischemia (murine)	Reduced NK cell cytotoxicity	Enhanced angiogenesis	[92]
Rat BM-MSCs	Hypoxia	Diabetic cardiomyopathy (Rat)	Upregulation of Bcl-2/Bax ratio, Inhibited expression and Activation of caspase 3	Anti-apoptotic Improved cardiac function	[93]
Human BM-MSCs	IFN-γ	Induced colitis (mice)	Increase in levels of IDO, iNOS Decrease in T cell proliferation, Decrease in inflammatory markers: TNF-a, IL-6, IL-17A	Decrease in disease score and diminished the severity of colitis	[94]
Murine BM-MSCs	IFN- γ	GvHD (murine)	GvHD mortality	Decrease in GvHD score after IFN- γ treatment at high concentrations	[95]
Human Wharton jelly MSCs	IFN- γ	Autoimmune encephalomyelitis (mice)	Increase in immunosuppressive factors: TGF-β, VEGF, HGF and IL-10, IL-4, Increase in T regulatory cells, Decrease in inflammatory factors- IL 17A	Decrease in disease score	[96]
Human BM-MSCs	IFN- γ	GvHD (Humanized mice)	T cell apoptosis and anergy	Reduced GvHD pathology and prolonged survival	[97]
UC-MSCs	TLR3 (poly I:C)	Trinitrobenzene sulfonate (TNBS)-induced colitis model (murine)	Reduced production of Th1/17 signature cytokines: IFN-γ, IL-17A, IL-21, and IL-23, Increased IL-10 production in the colon, Increased localization of Treg in colon Decreased infiltration of pathogenic Th1/17 subsets; Enhanced migration of UC-MSCs to inflammatory Sites, PGE2 mediated inhibition of mononuclear cell Proliferation	Enhanced immunosuppressive protective effect of UC-MSCs on experimental colitis	[98]
UC-MSCs	TLR3 (poly I:C) and TLR4 (LPS) priming	Dextran sulfate sodium-induced colitis model (murine)	TLR3 priming: inhibited T cell proliferation, Higher expression of IDO, TLR4 priming: Higher expression of proinflammatory cytokines- IL-6 and IL-8	Poly(I:C) primed UC-MSCs significantly ameliorated clinical and histopathological severity of DSS-induced colitis	[99]
Murine BM-MSCs	TLR1/2 (Pam3CSK4), TLR2 (PGN), TLR3 (polyI:C), TLR4 (LPS), TLR5 (flagellin), TLR2/6 (FSL-1), TLR7/8 (R848), TLR9-ODN 1826	Dextran sulfate sodium-induced colitis model (murine)	TLR3 priming: increased IDO expression	Poly(I:C)-treated MSCs attenuated the pathologic severity of DSS-induced murine colitis	[100]
Murine BM- MSCs	TLR3 (poly I:C) and TLR4 (LPS) priming	Experimental autoimmune encephalomyelitis (murine)	TLR 3 priming: reduced proliferation of CD3+ T cells, reduced \differentiation/activation of proinflammatory lymphocytes, Th1 and Th17 TLR4 priming: Increased CD3+ T-cell proliferation, induced Th1 and Th17 cells, Increased levels of proinflammatory cytokine IL-6	Pre-treatment of MSCs with poly(I:C) improved their therapeutic immunosuppressive abilities	[101]

### MSC licensing and activation

Mesenchymal stem cells are not spontaneously immunoregulatory, but they sense their microenvironment and perform accordingly i.e. either to induce immune tolerance or inflammation. For exerting the immunomodulatory functions, MSCs have to be primed with proinflammatory cytokines i.e. IFN-γ alone or in combination with TNF-α, IL-1α, IL-1β, or IL-17 [102, 103]. Most in vitro assays have indicated the importance of IFN-γ secreted by activated T cells for the commencement of MSC-mediated inhibitory mechanisms. However, MSCs might produce different responses under variable concentrations of IFN-γ and TNF-α. While lower concentrations of IFN-γ drive them to act as efficient antigen presenting cells (APCs) [104], higher concentrations inflict an inhibitory response [103]. The significance of an inflammatory environment for MSC immunosuppressive potential has been shown both in vitro and in vivo. The proinflammatory cytokines regulate a number of immunomodulatory soluble molecules produced by MSCs including IDO, NO, prostaglandin E2 (PGE2), TSG-6, TGF-β [105]. Under normal conditions, low levels of adhesion molecules are exhibited on the surface of MSCs. Pre-treatment of MSCs with appropriate concentration of proinflammatory cytokines, promotes immunosuppression, by enhancing the expression of cell adhesion molecules such as galectin-1, vascular cell adhesion molecule-1 (VCAM-1), chemokine ligands of C-C chemokine receptor type (CCR)-5 and C-X-C chemokine receptor type (CXCR)-3, that increase the cell–cell contact [106, 107]. In addition to this, proinflammatory cytokines also induce MSCs to secrete chemokine (C-X-C motif) ligand (CXCL)-9, CXCL10, and CCL2 (monocyte chemotactic protein-1), which are known to attract effector T cells [48]. Once MSCs and effector T cells come in contact, direct immunomodulation of T cells occurs via NO or FAS/FASL (FAS Ligand)-induced apoptosis [48, 108] and this response is further elevated when apoptotic T cells stimulate macrophage s to secrete TGF-β which in turn increase the regulatory cells.

A recent study has demonstrated decreased susceptibility of IFN-γ pre-treated cryopreserved MSCs to T cell-mediated apoptosis [109]. IFN-γ priming triggers immunosuppression by MSCs through up-regulation of B7-H1 molecule, also known as PD-L1, which acts as an inhibitory co-stimulatory molecule during immune responses [102]. Additionally, a recent study has reported that MSCs suppress T cell proliferation, seemingly through a cumulative effect of IFN-γ, TNF-α, and IL-17, leading to increased expression of inducible nitric oxide synthase (iNOS) [110]. Another study has revealed that IFN-γ promotes IDO expression in MSCs, which, consecutively suppresses the proliferation of effector T or B cells through the tryptophan pathway [111]. These findings indicate that pre-treatment of MSCs with pro-inflammatory cytokines enhance their immunoregulatory ability, which may prove valuable while evaluating them as a potential therapy.

### MSCs and toll-like receptors

Depending upon the type of TLR ligand involved in activation of MSCs, the MSCs become polarized en route for anti-inflammatory and pro-inflammatory phenotype [112]. This concept substantiates MSCs on one hand augmenting cell survival and function and on the other hand inhibiting inflammation and enhancing repair. Toll-like receptors are a set of an evolutionarily conserved family of receptors [113] with a tendency to identify molecular patterns associated with pathogens or “danger signals” associated with tissue injury. TLRs are present on immune as well as non-immune cells and are responsible for regulating both innate and adaptive immune responses [114]. Once a ligand binds to the respective toll-like receptor, a cascade of intracellular signalling pathways is instigated that direct activation of immune cells and release of cytokines and soluble mediators [115].

In context to TLR signalling, TLR-3 and TLR-4 activation of MSCs leads to augmented immunosuppression either due to IDO production induced via IFN-β and protein kinase R signalling [116] or due to regulatory T cell induction via notch signalling [117]. Another observation was made by Liotta et al. [118] shows the opposite effect with TLR3 and TLR4 binding directing downregulation of Jagged-1 which makes it impossible for MSCs to modulate T cell response. On similar lines, another study showed that TLR3/4 treated MSCs sustain the function of neutrophils by exerting anti-apoptotic effects which might trigger inflammatory disorders [119]. Few studies have shown that TLR4 and TLR3 can license MSCs differently i.e. TLR-3 priming induced anti-inflammatory phenotype of MSCs (MSC2) which produce IDO, PGE2, IL-4, IL-1RA and TLR4 priming induce pro-inflammatory type (MSC1) known for the production of IL-6, IL-8, TGF-β [112]. Stimulation of TLR9 has also been related to increasing immunosuppressive potential of MSCs together with a reduction in expression levels of TNF-α expression, increase in expression of TGF-β1 and adenosine [120]. TLR3 activation has also been shown to have protective effects on MSCs against NK cell killing and henceforth lead to successful and increased suppression of NK cells by MSCs [121]. Table 2 shows the compilation of studies attempted to monitor the effect of other TLRs on the immuno-modulatory property of MSCs. However, the studies highlighting the role of TLR in the immunomodulatory function of MSCs have presented mixed results and therefore extensive studies are required to elucidate the effects of TLR activation on MSCs.

### Drug interactions in vivo

Patients undergoing solid organ transplantation are usually medicated with a combination of IS drugs both pre and post-transplantation, in order to facilitate the graft outcome. IS drugs such as calcineurin inhibitors (Tacrolimus (TAC)), glucocorticosteroids and mTOR inhibitors, in combination improve the graft function by repressing the effector immune cell population thereby inhibiting the inflammatory responses [5], which is similar to the responses produced by MSCs [122]. Moreover, MSCs can weaken the negative effects produced by IS drugs on the immune system [123]. In view of that, various clinical trials concerning the use of MSCs to improve the outcome of graft have used MSCs as a supplemental therapy in addition to IS drugs. Of note, most of the clinical trials have not been able to reproduce the otherwise expected results. Perhaps, the immunoregulatory function of MSCs, which is dependent upon microenvironmental factors might also be influenced by the IS drugs. In patients suffering from end stage organ failure, hyperactivation of the immune system is evident [124, 125] and IS drugs resolve this by suppressing the pro-inflammatory cytokines [126, 127]. However, suppression of inflammatory mediators might adversely affect the licensing of functionally naive MSCs towards MSCs with anti-inflammatory function in solid organ transplant patients. A study conducted in an in vivo heart transplantation rat model examined the consequence of MSC administration in parallel to a common immunosuppressant cyclosporine A (CsA). Experiments suggested that while MSCs displayed immunosuppressive properties in vitro, this effect was reversed in presence of CsA which indicated a potential interaction between CsA and infused MSCs [58]. Similar findings were observed in another study in murine heart transplantation model using MSCs in combination with conventional immunosuppressants CsA, sirolimus and MMF [128]. Data from this study suggested that calcineurin inhibitors (CsA) prevented the activation of MSCs due to disrupted pro-inflammatory cytokine milieu which led to an aggravated anti-donor response while the combination of MSCs and MMF led to prolonged allograft survival. On contrary to this, a recent study in allogeneic mice model of skin transplantation has suggested induction of an alternatively “healing” phenotype of macrophages capable of producing high levels of IL-10 upon topical application of MSCs in combination with CsA [129].

Another form of immunosuppressants- glucocorticoids which act by blocking the biosynthesis of PGE2 [130] might interfere with MSC functionality as MSCs support synthesis of PGE2 which in return is responsible for inhibiting T cell proliferation [131]. A report on lung resident MSCs derived from human lung allograft patients demonstrated that both COX2 selective and non-selective COX inhibitor drugs block the immunosuppressive potential of MSCs on host immune cells [132]. These findings indicate that different drugs might behave differently as a result of interaction with MSCs in vivo. Acknowledging these findings, the IS drugs to be used together with MSCs should be selected very carefully.

## Other factors

Mesenchymal stem cells expansion has been established from multiple sources but their properties vary depending upon the site of their isolation [133]. Donor heterogeneity is yet another concern while considering the use of MSC therapy [134]. Age of donor may influence the therapeutic value of MSCs as MSCs derived from old donors have diminished proliferation potential [135] along with an altered membrane glycerophospholipid composition [136]. Moreover, lack of standardized isolation and expansion protocols affect the qualitative properties of MSCs to a great extent. In view of the ongoing trials, there is a lot of variation in the number of cells used for infusion, number of doses, infusion time points and transfusion patterns, which might be a reason behind inconsistent outcomes (Table 1). Therefore, immediate attention is required to deal with these issues for improving the overall therapeutic efficacy and for facilitating the utilization of MSC therapy.

## Future perspectives

The chief objective of applying MSCs as maintenance immunosuppressive therapy is to augment allograft acceptance and function. Ongoing research has suggested a key role of the microenvironment in defining the fate of MSC therapy. In light of these stimulating findings, we hereby propose different approaches for MSC modifications that can contribute to the success of this therapy.

### MSC modification: preconditioned or genetically modified

To overcome the current limitations, MSCs after isolation can be cultured or pre-conditioned in hypoxic conditions, so as to maintain a native healthy profile which enhances their immunomodulatory and regenerative capacity. Further, preconditioning of MSCs with proinflammatory factors could also help in abolishing their behavioural heterogeneity [137] thus making them appropriate for application in transplant patients. Some preclinical studies have also demonstrated that genetic modification or engineering of MSCs could also benefit in disease management [138–140]. Targeted delivery of MSCs with triple engineering (P-selectin glycoprotein ligand-1 (PSGL-1)/Sialyl-Lewis(x) (SLeX)/IL-10) has shown superior therapeutic function over the unmodified MSCs in a murine model of autoimmune encephalomyelitis (EAE) [141]. Most recently, MSCs engineered with Etanercept, a TNF-α blocker, were used effectively in a mice model of collagen-induced arthritis [142]. On similar lines, there exist several cytokines, growth factors, TLR agonists which can be used individually or in combination to treat MSCs, for encouraging their therapeutic efficacy.

### MSCs: for supplemental therapy

Mesenchymal stem cells have long been used in combination with IS drugs for immune-mediated conditions. However, in most trials IS drugs are administered prior to MSC infusion, which might lead to an altered cytokine profile that MSCs experience in vivo resulting in their poor efficacy. Therefore, optimization of timing, number of cells per dose, number of doses and route of administration would be of immense advantage while considering the use of MSCs for immune-mediated conditions.

### MSCs: for personalized therapy

**Fig. 1:**
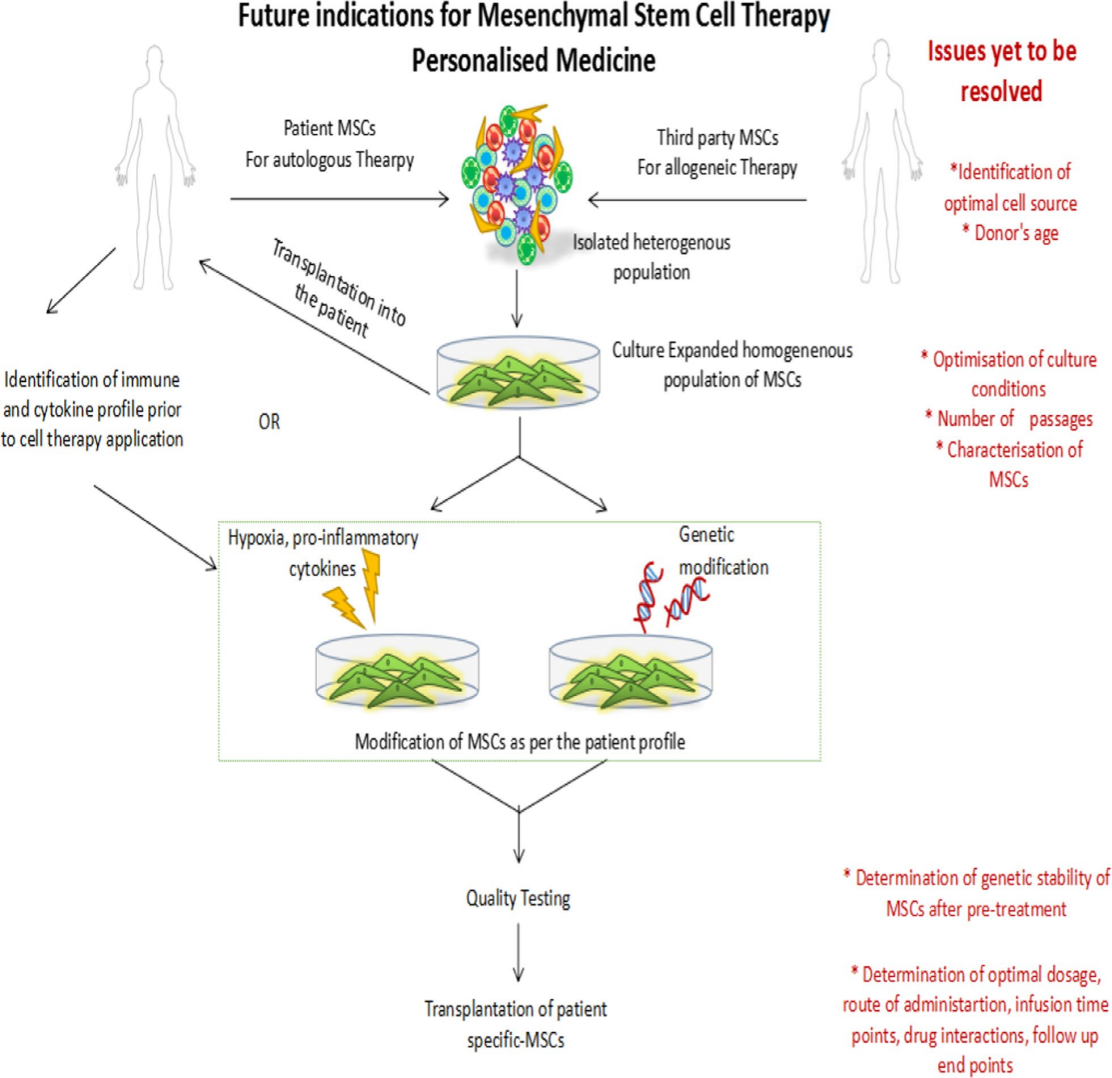
Approaches for modification of mesenchymal stem cell therapy for application in patient-based trials. The primary approach is to identify the optimal cell source and conditions to be maintained for their expansion, followed by the functional characterisation of these cells through in vitro and animal studies. Secondly, determination of the dosage, route of administration and infusion time points through animal studies and pilot trials is crucial. Further, before applying MSCs for patient care, determination of patient profile is important, as this will allow us to establish whether these cells require any modifications to enhance their therapeutic efficacy or not. Finally, autologous or allogeneic MSCs isolated from an optimal source and expanded in favourable culture conditions can either be applied directly to the patients or can be modified as per the patient’s profile and then applied to the patients after assessment of their genetic stability

Clinical trial outcomes have emphasized the concept of “licensing”, which is easily controllable in vitro, but, remains to be a challenge in in vivo condition. In patients, the inflammatory milieu is variable depending upon the immune disorder. This variation in personal microenvironment is responsible for altering the behaviour of infused MSCs. To ensure the success of MSC therapy, it, therefore, becomes necessary to study and understand the signalling molecules and cellular interactions in the prospective microenvironment of a patient. It is the heterogeneity in MSC profile based on isolation and culture protocols and the patient factors which substantiates the need for personalized medicine. Thus it would be beneficial to identify the cytokine and immune status of patients prior to MSC application. Patient population likely to benefit may be given the MSC therapy without any modification while for others, individualization of MSC therapy using either genetically modified or pre-conditioned MSCs may prove to be beneficial (Fig. 1).

## Conclusion

In past few years, a plethora of studies have theorized and substantiated the immunosuppressive potential of MSCs. Data from clinical trials have assured the safety of MSC based therapies in organ transplant patients. However, the results in terms of efficacy have not been satisfactory which insinuates the need to authenticate these findings further, before implementing this as a global therapy. Although the therapeutic efficacy of engrafted MSCs has not been fully established the microenvironmental cues regulating their plasticity are well indicated, which, if modulated, can result in enhanced efficaciousness. The capability of MSCs to respond differently to variable levels of inflammation, cytokines and immunosuppressive agents have drawn the attention towards their functional plasticity. Understanding and translation of MSC-plasticity mediated immune-regulation can help improvise the foundation of MSC therapy. Moreover, as a part of personalized medicine, it would be beneficial to standardize the protocols for pre-conditioning or genetically modifying MSCs as per the patient’s need to further enhance the applicability and success of these cellular therapies which in future may substitute the current drug therapies.

## Abbreviations

IS drugs: immunosuppressive drugs
MSC: mesenchymal stem cell
APC: antigen presenting cells
TLR: toll-like receptor
BM: bone marrow
UC: umbilical cord
MHC: major histocompatibility complex
IFN-γ: interferon-γ
Tregs: regulatory T cells
Bregs: regulatory B cells
DC: dendritic cell
MEM: MSC-educated macrophages
IL-10: interleukin-10
NK: natural killer cells
TGF-β: transforming growth factor
HGF: hepatocyte growth factor
NO: nitric oxide
HO-1: heme oxygenase-1
IDO: indoleamine 2,3-dioxygenase
TRAIL: TNF-related apoptosis-inducing ligand
PD-L1: programmed death ligand 1
BM-MSCs: bone marrow derived MSCs
UC-MSCs: umbilical cord derived MSCs
PBMC: peripheral blood mononuclear cell
Tx: transplantation
TAC: tacrolimus
ATG: anti-thymocyte globulin
MMF: mycophenolate mofetil
CNI: calcineurin inhibitor
MEP: methylpremnisolone
CsA: cyclosporine A
HIF: hypoxia inducible factor
HIF-1α: HIF-1 alpha
HIF-2α: HIF-2 alpha
KLF4: Kruppel like factor 4
OCT4: octamer-binding transcription factor 4
C-MYC: v-myc avian myelocytomatosis viral oncogene homolog
PGE2: prostaglandin-E2
DSS: dextran sulfate sodium
VCAM-1: vascular cell adhesion molecule-1
CCR5: chemokine ligands of C-C chemokine receptor type-5
CXCR3: C-X-C chemokine receptor type-3
CXCL9: chemokine (C-X-C motif) ligand-9
CXCL10: chemokine (C-X-C motif) ligand-10
CCL2: monocyte chemotactic protein-1
iNOS: inducible nitric oxide synthase
IFN-β: interferon-β
PSGL-1: P-selectin glycoprotein ligand-1
SLeX: Sialyl-Lewis(x)
EAE: autoimmune encephalomyelitis
